# Simultaneous Damage of the Cingulate Cortex Zone II and Fronto-Striatal Circuit Causes Prolonged Selective Attentional Deficits

**DOI:** 10.3389/fnhum.2021.762578

**Published:** 2021-12-24

**Authors:** Riho Nakajima, Masashi Kinoshita, Mitsutoshi Nakada

**Affiliations:** ^1^Department of Occupational Therapy, Faculty of Health Science, Institute of Medical, Pharmaceutical and Health Sciences, Kanazawa University, Kanazawa, Japan; ^2^Department of Neurosurgery, Faculty of Medicine, Institute of Medical, Pharmaceutical and Health Sciences, Kanazawa University, Kanazawa, Japan

**Keywords:** cingulate cortex, zone II, brain tumor, right cerebral hemisphere, selective attention

## Abstract

Selective attention is essential for successful cognitive performance. Although several brain areas are known to be involved in selective attention, damage to some of these areas does not necessarily cause attentional deficits. In the current study, we hypothesized that damage to specific parts of the right cerebral hemisphere, especially the cingulate cortex (CC), causes prolonged selective attentional deficits, and examined the influence of focal brain damage on selective attention. We recruited 36 patients with right cerebral hemispheric WHO grade 2 and 3 brain tumors who underwent surgery. We assessed selective attention over time from pre-operation to 3 months postoperatively using the cancelation test and color Stroop test, and calculated the percentage of deficit. Additionally, two types of imaging analyses were performed: voxel-based lesion symptom mapping (VLSM) and multiple logistic regression analysis, to reveal related brain regions for selective attention. Consequently, we found that the CC and deep part of the middle frontal gyrus were associated with deficits in selective attention via VLSM. Using multiple logistic regression analysis, the CC zone II at the cortical level (*p* < 0.0001) and the fronto-striatal tract (FST) at the subcortical level (*p* = 0.0079) were associated with attentional deficit among several regions identified in the VLSM. At 3 months postoperatively, selective attention was impaired in patients who underwent resection of these regions. Moreover, only patients with simultaneous damage of the CC zone II and FST had prolonged attentional deficits until the chronic phase. Our results suggest that the right CC zone II and FST are critical areas for the selective attentional networks.

## Introduction

The ability to select relevant information for our environment is considered the basis of all cognitive functions (Rosenberg et al., 2016; Kim and Kastner, 2019), and is frequently influenced by neurological/neurosurgical diseases. Selective attention, which is a core component of attention, can direct specific modalities, spaces, and features at the expense of other disturbances (Mueller et al., 2017). To do so, they need to direct attention voluntarily to appropriate stimuli toward the target (Corbetta and Shulman, 2002; Buschman and Miller, 2007; Mok et al., 2019). Additionally, selective attention requires inhibition of others’ interference to direct attention toward a specific one (Mueller et al., 2017).

Several brain areas are known to be involved in selective attention, such as the dorsolateral and inferior prefrontal cortices; medial prefrontal cortices, especially the dorsal anterior cingulate cortex (CC); orbito-frontal cortex; inferior parietal lobule; and superior temporal cortex (Hopfinger et al., 2000; Schulz et al., 2011; Mansouri et al., 2020). At the subcortical level, the frontoparietal network, namely the superior longitudinal fasciculus (SLF) and fronto-striatal circuit, are known to be involved in selective attention (Thiebaut de Schotten et al., 2011; Marshall et al., 2015; Naaijen et al., 2018). Among these, the anterior CC plays a central role in selective attention, specifically voluntary attentional control (Zhang et al., 2017; Mok et al., 2019), and it monitors appropriate action to achieve high-level goals with merging cognitive, sensorimotor, and incentive signals (Botvinick et al., 1999; Schulz et al., 2011). Regarding lateralization, although some of the past research emphasized the importance of the right hemisphere for attention function, there is no consensus to date (Heilman and Van Den Abell, 1980; Posner and Petersen, 1990; Nobre et al., 1997; Corbetta, 1998; Corbetta and Shulman, 2002). However, previous studies on patients with brain damage demonstrated that attention functional deficits tend to be found after damage to the right side, rather than the left side (Nobre et al., 1997; Wang et al., 2016; Agosta et al., 2017; Spaccavento et al., 2019). Additionally, participants with a larger tract volume of the right SLF than those on the left side had a higher ability to regulate selective attention (Marshall et al., 2015). These results might indicate that the right hemisphere plays a critical role in attention function, even though selective attention is governed by the bilateral hemispheres.

In this context, lesion studies, including brain tumor surgery, can provide insight into changes that might occur in the absence of a specific brain region. For instance, in patients who underwent cingulotomy for intractable pain or mental illness, selective attention was damaged following surgery, whereas other neuropsychological functions such as general intelligence, language, memory, and motor skills were mostly intact (Lenhard et al., 2005; Yen et al., 2009). This is also evidence that the anterior CC plays a critical role in the selective attentional network. However, attentional deficits are not necessarily elicited, even though a part of the anterior CC is damaged locally (Baird et al., 2006).

In the current study, we hypothesized that damage to specific parts of the right cerebral hemisphere, especially the CC, causes prolonged selective attentional deficits. We then examined the selective attention of patients with focal brain damage in right cerebral hemispheric brain tumors. Here, we used two types of assessment of selective attention, the cancelation test and color Stroop test, but the aspects assessed by each test are slightly different. In the cancelation test, participants are required to focus their attention on a specific stimulus intentionally while actively ignoring distractor stimuli (Lezak, 1995; Casco et al., 1998; Trevino et al., 2021). In contrast, the Stroop test is an assessment of selective attention involving attentional control or inhibition (Treisman and Fearnley, 1969; Graf et al., 1995). Consequently, we found that simultaneous damage of the anterior part of the anterior CC zone II and FST causes a prolonged deficit of selective attention, and these regions might be critical areas in the selective attention system.

## Materials and Methods

### Participants

**FIGURE 1:**
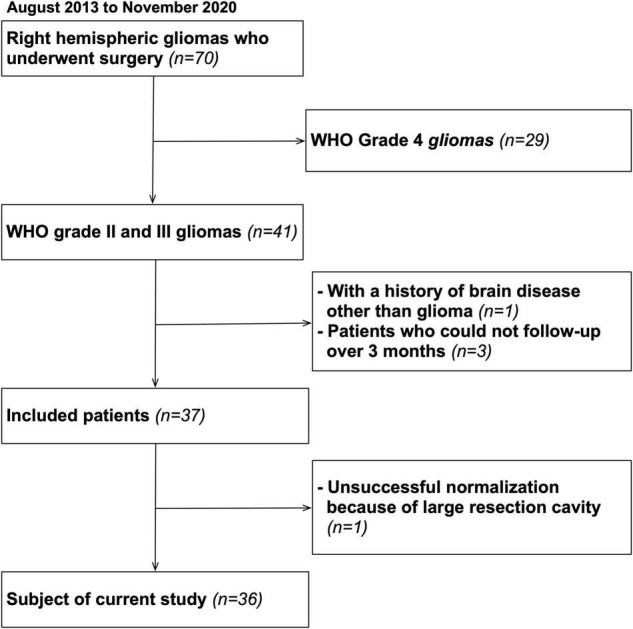
Flow chart of the inclusion criteria.

A total of 70 patients undergoing surgery for resection for right cerebral hemispheric brain tumors in Kanazawa University hospital from August 2013 to November 2020 were enrolled. Among them, 36 WHO grade II and III brain tumors completed serial neuropsychological examination until postoperative 3 months without recurrence, and they matched inclusion criteria of our cross-sectional study (Figure 1). A summary of patient demographic and clinical characteristics are shown in Table 1. Written informed consent was obtained from all patients. This study was performed according to the guidelines of the Internal Review Board of Kanazawa University and was approved by the Medical Ethics committee of Kanazawa University (approval numbers 1,797 and 3,160).

**TABLE 1:** Demographic and clinical characteristic of participants.

Factors	Value
**Age**	
Mean ± SD	48.7 ± 13.0
Range	21–72
**Sex**	
Male	23
Female	13
**WHO grade**	
II	22
III	14
**Histology**	
Diffuse astrocytoma	12
Oligodendroglioma	10
Anaplastic astrocytoma	5
Anaplastic oligodendroglioma	9
**IDH-1**	
Mutant	32
Wild type	3
ND	1
**1p19q**	
Codeletion	18
Intact	15
ND	3
**Operative method**	
Awake surgery	36
General anesthesia	0
**Preop tumor volume**	
Mean ± SD (cc)	39.2 ± 41.7
**Receiving chemotherapy**	
Yes	16
No	20
**Receiving radiotherapy**	
Yes	6
No	30

*IDH, isocitrate dehydrogenase; ND, not detected; SD, standard deviation.*

### Assessment of Attentional Function

All patients underwent assessment for selective attention at pre-operation, post-operative 1 week and 3 months postoperatively. We used two types of assessment for selective attention: the letter cancelation test and color Stroop test. In the letter cancelation test, patients were asked to mark the specified character (e.g., “ka,” the *kana* character) as quickly and accurately as possible from a total of 114 *kana* syllabary characters presented on A3-size paper (Lezak, 1995; Commition-of-Brain-Function-Test, 2006). The percentage of correct answers was recorded, representing score of selective attention. To note that, letter cancelation test as a part of “Clinical assessment for attention” (Commition-of-Brain-Function-Test, 2006) used in current study is different from assessment of unilateral spatial neglect, such as “Behavioral inattention test” (Wilson et al., 1999). Selective attention with cognitive control can be assessed via the color Stroop test. The Stroop effect refers to the phenomenon that two simultaneously incoming visual information interfere each other (Stroop, 1935). The color Stroop test is useful for assessing the ability to inhibit stereotyped behavior or unwanted information and to control focused attention (Perret, 1974). The test consists of presenting the names of colors (e.g., red or green) written in different colored ink (e.g., “red” is printed in yellow ink or “green” is printed in blue ink). Patients are asked to specify the color of the ink instead of reading the words as quickly as possible. If the patient made a mistake (e.g., reading the color), he/she was made to correct it immediately. The overall response time was used as the score of the Stroop test. All assessments were performed in a noise-controlled room by a trained occupational therapist. These two tests were part of a neuropsychological assessment over time for the normal medical practice of brain tumors. Usually, patients undergo these examinations for 1 h. Considering the patients’ concentration, we performed an assessment of selective attention for about 10 min from the start of the examination. It took approximately 5 min for the letter cancelation test and the Stroop test.

### Magnetic Resonance Images and Lesion Mapping

Structural MR images were acquired postoperatively (every 3 months) as standard care. The images were acquired using conventional high-resolution three-dimensional (3D) T1-weighted inversion recovery-prepared fast spoiled gradient-echo sequence, performed with a repetition time 6.2 ms, echo time 2.4 ms, slice thickness 1.4 mm, field of view 220 mm, matrix size 256, flip angle 15°, inversion time 700 ms, and number of signals averaged 1 on a 3.0 T MRI (Signa HDxt, GE Healthcare, Milwaukee, WI, United States). Each image was conformed to the MNI template using SPM12,^1^ which was implemented in MATLAB.^2^ Normalization was performed using *cost function masking* (Brett et al., 2001), which is commonly used in the normalization of brain with lesions or resection cavities, in order to avoid registration errors due to the presence of lesion-related abnormal signals (Andersen et al., 2010; Herbet et al., 2016). Each individual MR image transformed into an MNI space was checked to determine the accuracy of transformation by comparing anatomical landmarks such as the sulci and brainstem. The resection cavities were then drawn manually using MRIcron software^3^ for each patient. Reconstruction was firstly performed by RN and then systematically checked by a neurosurgeon (MK).

### Statistical Analysis

#### Behavioral Data

All row test scores [percentage of correct answers (%) in letter cancelation test and overall response time (sec) in Stroop test] were converted to Z-scores using the mean and standard deviations (SD) of the patients’ score at baseline (Nakajima et al., 2019). Here, a deficit was recorded if the degree of decline of the Z-score was -1.65 or less as previously reported (van Geemen et al., 2014; Herbet et al., 2015; Nakajima et al., 2019). Patients were classified as un-impaired if both test scores were normal and as impaired if one or either of the assessments declined. We calculated percentage of deficit and compared it between groups using Chi-square test. We statistically analyzed the patients’ data with non-parametric analyses because the data did not exhibit a normal probability distribution.

#### Voxel-Based Lesion-Symptom Mapping

To identify regions related to total score of attention, the VLSM was performed using NPM software in the MRIcron package (see text footnote 3) as previously described (Bates et al., 2003). The analysis was performed in binary data; whether participants had attentional deficit or did not. To minimize possible outlier effects, the analysis was performed only on voxels resected by surgery in at least three patients (Campanella et al., 2014). Parametric *t*-tests were used to generate the statistical maps. To control for false positives (type I error) due to multiple comparisons, we systematically corrected the resulting statistical maps using the false discovery rate procedure with a threshold of *p* = 0.05 (Menyhart et al., 2021). Significant differences between the severity level and voxels with and without lesions were identified and presented as Z-scores on the MNI coordinates.

#### Univariate and Multiple Logistic Regression Analysis

To reveal the critical region for attentional deficit among several significant regions identified by the VLSM, univariate and multiple logistic regression analyses were performed. In cortical level, the resected volume of each gyrus was calculated by following steps. First, each resection cavity map was overlaid on an automated anatomical labeling (AAL) template using MRIcron software (see text footnote 3). Then, the MRIcron software automatically computed the number of voxels that overlapped cortices. Similarly, the resected amounts of white matter tracts at the level of the VLSM significant region were estimated using the following steps (Supplementary Figure 1). In this procedure, we used the white matter atlas, which tract probabilities were over the 0.50 threshold, provided by Rojkova et al. (2016). Each white matter tract was superimposed on the significant regions of the VLSM, and the extent of white matter tracts damaged by the significant regions of the VLSM was identified. Then, each resection cavity map was overlaid. Finally, MRIcron software was used to compute the number of voxels that overlapped white matter tracts in the resection cavity at the level of the VLSM significant region. We then used statistical analysis software (JMP, version 14.3.0; SAS Institute, Inc., Cary, NC, United States) to perform Wilcoxon test and multiple logistic regression analysis with stepwise methods to analyze relationships between the behavioral data and the attentional deficit in each gyrus and white matter tract. Descriptive variables was resected volume of cortices and white matter tracts in relation to the VLSM positive region, while the objective variables was presence of attentional deficit.

## Results

### Anatomical and Behavioral Data

**FIGURE 2:**
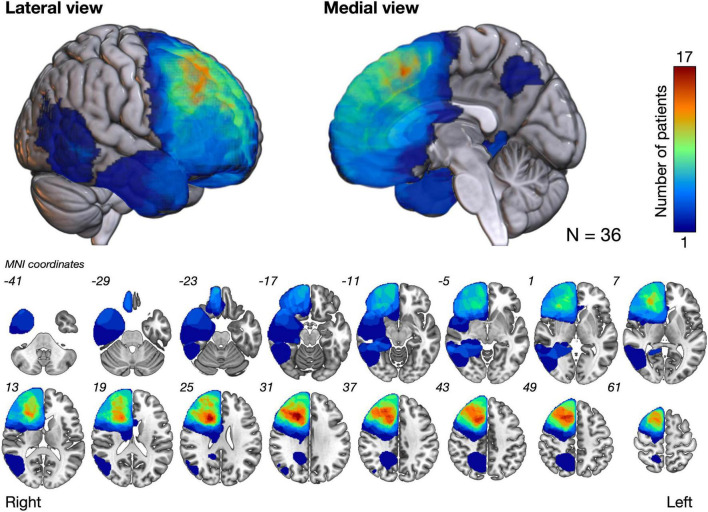
The overlap map of resection cavities shows that the deep part of the medial prefrontal cortex had the greatest degree of overlap (*N* = 17). MNI, Montreal neurological institute.

**FIGURE 3:**
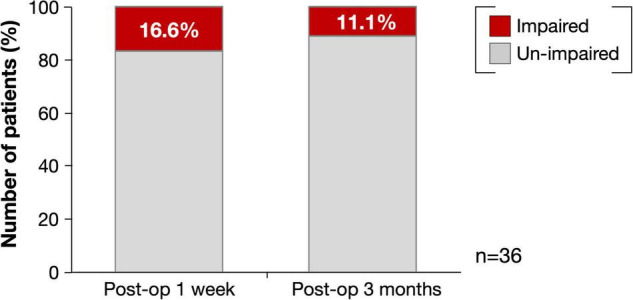
Percentage of deficit for selective attention at postoperative 1 week and 3 months. Red, impaired patients (Z ≤ −1.65); Light gray, un-impaired patients.

Figure 2 shows the overlap maps of all the resection cavities (*n* = 36). The greatest overlap maps of resection cavities (*N* = 17) were in the deep part of the medial prefrontal gyrus, which was on the course of the fronto-striatal tract (FST), frontal aslant tract, and cingulate tract. Selective attention was impaired (Z ≤ −1.65) in 16.6% (*N* = 6) of patients during the acute phase and in 11.1% (*N* = 4) of patients at 3 months postoperatively (Figure 3 and Supplementary Table 1). Among the impaired group, patients with impaired letter cancelation test and Stroop test were as follows: at 1 week postoperatively, 83.3 and 33.3%, respectively; at 3 months postoperatively, 100 and 50.0%, respectively (Supplementary Figures 2, 3).

### Regions Related to Selective Attentional Deficit

**FIGURE 4:**
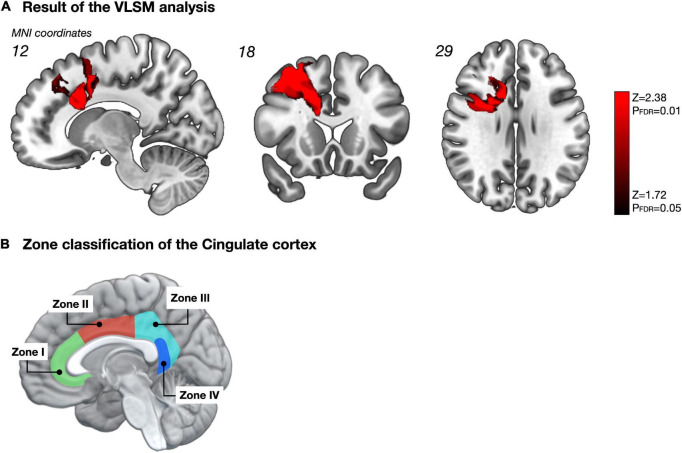
Impact of resected region on selective attention **(A)**. The statistical map obtained from the voxel-based lesion-symptom mapping (VLSM) shows only significant voxels with a false discovery rate-controlled threshold (*p* = 0.05; z = 1.72). Among these significant regions, the cortical area that showed the highest correlation with selective attention was the middle CC, namely zone II of the CC, according to multiple logistic regression analysis with Stepwise method **(B)**. The figure B was made based on Tate et al. (2011). Permitted by J. Neurosurg.

We analyzed the spatial locations related to attentional deficits using the VLSM. One week postoperatively, the VLSM for the attentional deficit revealed that the largest clusters of significant voxels were in the CC and middle frontal gyrus (cluster size, 33,280 voxels; Z_max_ = 3.49; Figure 4A). We then investigated the region most responsible for attentional deficits among several VLSM-positive regions. First, we analyzed cortical areas that overlapped with the VLSM-positive region. The following gyri were identified at the cortical level: the precentral, frontal superior, frontal middle, frontal inferior operculum, frontal inferior triangularis, supplementary motor area, frontal superior medial, insula, anterior CC, and middle CC. In univariate analysis, we divided the patients into two groups according to the presence or absence of attentional deficit, as mentioned above, and compared the resected volume of each gyrus between groups (Table 2). Consequently, the resected volume of the supplementary motor area (*p* = 0.045) and middle CC (*p* = 0.035) were significantly higher in the impaired group. However, when considering multiple comparisons, these results were not statistically significant. Next, to select the optimal dependent variables, we first performed a stepwise analysis. Among all explanatory variables, the resected volume of the frontal middle gyrus, supplementary motor area, anterior CC, and middle CC were selected by a stepwise method, and only the middle CC, which corresponds to the CC zone II (Figure 4B; Tate et al., 2011) was associated with attentional deficit (*p* < 0.0001; Table 2). Similarly, the arcuate fasciculus, cingulate tract, frontal aslant tract, FST, SLF-dorsal segment, and ventral segment were found to overlap in the VLSM positive region at the subcortical level; in univariate analysis, all tracts correlated significantly with acute attentional deficit (Table 3). Furthermore, multiple logistic regression analysis showed that only FST was associated with selective attention.

**TABLE 2:** Relationship between resected volume of cortical area and attentional deficit.

Cortical structure (AAL’s label)	Postop 1 week (*p*-value)	
	Univariate analysis	Multiple logistic-regression analysis
Precentral	0.13	–
Frontal superior	0.076	–
Frontal middle	0.016*	0.99
Frontal inferior operculum	0.066	–
Frontal inferior triangularis	0.12	–
Supplementary motor area	0.045*	0.97
Frontal superior medial	0.23	–
Insula	0.49	–
Anterior cingulate cortex	0.21	0.98
Middle cingulate cortex	0.035*	<0.0001****

As for univariate analysis, Wilcoxon test was performed in each gyrus. Before performing multiple logistic-regression analysis, we targeted cortical and subcortical regions using a Stepwise method. Minus (−) indicates the factor which was not chosen by Stepwise methods as possible explanatory variable. *p < 0.05, ****p < 0.0001.

**TABLE 3:** Relationship between resected volume of white matter tracts and attentional deficit.

White matter tracts	Postop 1 week (*p*-value)	
	Univariate analysis	Multiple logistic-regression analysis
Arcuate fasciculus—long segment	0.0024**	–
Cingulum	0.0024**	–
Frontal aslant tract	0.0014**	–
Fronto-striatal tract	0.0010**	0.0079**
Superior longitudinal fasciculus—dorsal segment	0.0015**	–
Superior longitudinal fasciculus—ventral segment	0.0069**	–

As for univariate analysis, Wilcoxon test was performed in each gyrus and tract. Before performing multiple logistic-regression analysis, we targeted cortical and subcortical regions using a Stepwise method. Minus (−) indicates the factor which was not chosen by Stepwise methods as possible explanatory variable. **p < 0.01.

**FIGURE 5:**
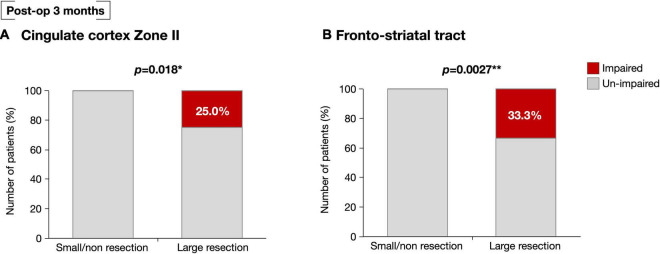
At postoperative 3 months, selective attention was impaired significantly high possibility in patients with large resection of the CC zone II **(A)**. Similarly, selective attention was significantly impaired in those with greater resection of the FST at the level of deep medial prefrontal cortex **(B)**. *p < 0.05, **p < 0.01.

**FIGURE 6:**
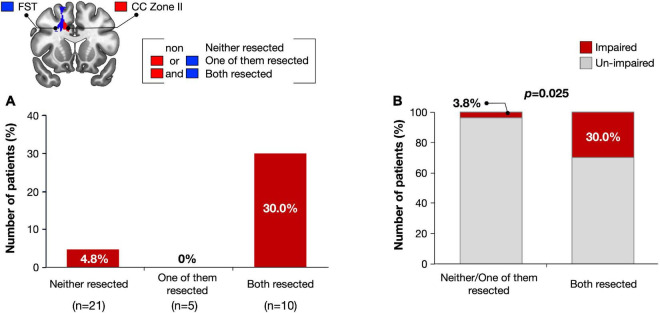
Number of impaired patients were compared among three groups; Subjects with both FST and CC Zone II resected, subjects with only one of them resected, and subjects with neither of them resected **(A)**. Only when the CC zone II and FST were damaged simultaneously, attentional deficit was remained with significantly high possibility **(B)**.

We then examined the influence of resection of the VLSM-positive region on selective attention at 3 months after surgery. Using cluster analysis, resected volumes of CC zone II and FST were divided into two clusters, namely the small/non-resection group and the large resection group (Supplementary Figure 4). We compared selective attention at 3 months postoperative between the two groups and found that the deficit rate of the large resection group was significantly higher in both the CC zone II and FST (*p* = 0.018 and *p* = 0.0027, respectively) (Figure 5). The following *post hoc* analyses were then performed to investigate whether the FST and CC can cause an attentional deficit at 3 months postoperatively, even if they are single injuries. All participants were then divided into three groups according to the amount of CC zone II and FST resected: “neither resected,” “only one of them resected,” and “both resected.” The percentages of patients with impaired selective attention were 4.8, 0, and 30%, respectively (Figure 6A). Finally, we compared attentional deficit at 3 months between the neither/one of the resected and resected groups and found that the impaired ratio was significantly higher in both the CC zone II and FST resected groups (Figure 6B, 3.8 and 30.0%, respectively, *p* = 0.025). Significant differences between these two groups were not found in other neurological and cognitive functions except for selective attention, including movement, sensation, general cognitive function, working memory, verbal fluency, visuospatial cognition, and emotion recognition (Supplementary Table 2).

## Discussion

In the present study, we investigated the regions that play a critical role in selective attention in right cerebral hemispheric brain tumors. We found that simultaneous damage to both the right CC zone II and FST cause a prolonged deficit in selective attention. Therefore, CC zone II and FST may be considered as key areas for selective attentional networks.

The central roles of the CC are high-level processing and selection, such as emotional function, sense of pain, and cognitive control and/or goal-directed attention (Shackman et al., 2011; Bubb et al., 2018; Touroutoglou et al., 2019). Specifically, zone II might be a key structure for selective attention, since damage to the region caused prolonged deficits in selective attention in the current study. Previously, it was known that CC is one of the central areas of selective attention. For instance, previous neuroimaging and neurophysiological studies and direct electrical stimulation during brain tumor surgery revealed the related brain regions such as the frontal lobe including the CC, dolso-lateral prefrontal cortex, and inferior frontal gyrus, in the Stroop test (Goethals et al., 2004; Wager et al., 2013; Song and Hakoda, 2015; Zhang et al., 2017; Heidlmayr et al., 2020). In particular, the CC plays an important role in tasks that require selective attention with cognitive control, such as the Stroop test (Wager et al., 2013; Heidlmayr et al., 2020). On the other hand, it is not common knowledge that CC zone II plays a central role in selective attention. To support our results, a meta-analysis indicated that the right hemispheric regions, including the middle part of the CC, play a role in inhibitory control during selective tasks, independent of the task types (Zhang et al., 2017). Moreover, some previous reports revealed that the CC plays important role in selective attention, and its location of significant region on neuroimaging results corresponds to the CC zone II (Silton et al., 2011; White et al., 2018). Beyond attentional function, some authors have revealed the importance of zone II within the CC, corresponding to the middle CC for various kinds of function. For instance, a meta-analysis including 380 activation foci from 192 studies found that the anterior part of the middle CC was the unique region that activates every functional domain (Shackman et al., 2011). Therefore, the anterior part of the CC zone II is considered a hub of cognitive and behavioral control, in particular selective attention (Shackman et al., 2011; Touroutoglou et al., 2019).

In the current study, the CC as well as the FST played a critical role in selective attention. Previously, the fronto-striatal circuit was known to play a critical role in the attentional network (Arnsten and Rubia, 2012; van Ewijk et al., 2012). Specifically, previous DTI studies revealed that abnormality of structural connectivity in the FST is associated with attentional deficits in patients with attention-deficit hyperactivity disorder (ADHD) (Silk et al., 2009; Konrad et al., 2010; van Ewijk et al., 2012). Moreover, a meta-analysis revealed that the fronto-striatal circuit underlies response inhibition, such as the Go/No-Go task (Zhang et al., 2017).

Interestingly, our results revealed that simultaneous damage to the FST and CC zone II caused a prolonged attentional deficit over 3 months postoperatively. Recently, it has been shown that there is functional connectivity between the fronto-striatal circuit and anterior CC (Naaijen et al., 2018; Hammes et al., 2019). Among the fronto-striatal circuits, reduced functional connectivity between the anterior CC and the striatum may cause an attentional deficit in ADHD (Hammes et al., 2019). Generally, deficits in neurological/neuropsychological function following brain tumor surgery tend to be temporary and have the ability to recover (Nakajima et al., 2017). Particularly, functions that are governed by extensive brain networks, such as attention, are more likely to recover, even though some of them are damaged (Price and Friston, 2002). Considering all these facts, simultaneous damage of two key areas might result in irreversible damage.

There are some potential methodological limitations. First, we were able to use only a limited number of assessments for attention since we performed these tests as a part of standard care during the perioperative period. In line with this, we focused on right hemispheric brain tumors in the current study, since the right hemisphere is considered to play an important role in the attentional network (Agosta et al., 2017; Spaccavento et al., 2019). Another reason for focusing on the right side is that it is difficult to assess attentional function of right and left lesioned patients using same test battery, since the cause for declining test score of left hemispheric brain tumors are sometimes different from that of right side (e.g., aphasia or alexia) (Spaccavento et al., 2019). Second, since the purpose of the current study was to investigate the influence of the resected area by investigating the relationship between focal brain resection and function, the attentional score should be based on the baseline. Therefore, some of the patients in the normal group had impairment when their scores were compared with those of the age-matched controls (Supplementary Table 1, asterisk). Finally, radiation therapy may induce delayed decline of cognitive function (Tanriverdi et al., 2016). Recent study has demonstrated that the impact of irradiation to white matter appears in 6 months after irradiation and cognitive function declines much later (Chapman et al., 2016). Considering these facts, the possibility that radiation therapy influenced attentional deficit in our patient group was low since the observation period in current study was 3 months. Moreover, there was no significant difference in the attentional scores of the six patients who received radiotherapy compared to those who did not in our patient group (Supplementary Figure 5). Further study will be required using a large number of patients with well controlled group in the future.

## Conclusion

We revealed that zone II of the right CC and FST may be one of critical area in the selective attentional network, and concurrent damage of these regions might cause long-term deficit of selective attention. Lesion studies of patients who underwent focal brain resection are considered a unique method to understand key areas of the brain network.

## Data Availability Statement

The original contributions presented in the study are included in the article/Supplementary Material, further inquiries can be directed to the corresponding author/s.

## Ethics Statement

This study was performed according to the guidelines of the Internal Review Board of Kanazawa University and was approved by the Medical Ethics committee of Kanazawa University (approval numbers 1,797 and 3,160). The patients/participants provided their written informed consent to participate in this study.

## Author Contributions

MN and RN: conception and design, drafting article. RN: acquisition of data. RN and MK: analysis and interpretation of data. MN: study supervision. All authors critically revising the article and reviewed final version of the manuscript and approved it for submission.

## Conflict of Interest

The authors declare that the research was conducted in the absence of any commercial or financial relationships that could be construed as a potential conflict of interest.

## Publisher’s Note

All claims expressed in this article are solely those of the authors and do not necessarily represent those of their affiliated organizations, or those of the publisher, the editors and the reviewers. Any product that may be evaluated in this article, or claim that may be made by its manufacturer, is not guaranteed or endorsed by the publisher.

## Footnotes

1
http://www.fil.ion.ucl.ac.uk/spm/software/spm12/

2
http://www.mathworks.com/products/matlab/

3
http://www.mccauslandcenter.sc.edu/mricro/mricron/

## Supplementary Material

Supplementary Figure 1 A schema for calculating resected volume of white matter at the level of deep medial prefrontal cortex, namely voxel-based lesion-symptom mapping (VLSM) positive region.

Supplementary Figure 2 Impaired ratio of selective attention in each test at post-op 1 week **(A)** and 3 months **(B)**. Red, impaired (Z ≤ −1.65); Gray, un-impaired (Z > −1.65).

Supplementary Figure 3 Details of selective attention in each test at post-op 1 week **(A)** and 3 months **(B)**. Wilcoxon test is used to compare the groups. Red line, mean value; Blue line, standard deviation.

Supplementary Figure 4 Resected volume of the middle CC **(A)** and fronto-striatal tract **(B)** were divided into two groups using cluster analysis. We named cluster 1 as small/non-resection group, and cluster 2 as large resection group. Red, mean value; blue, standard deviation.

Supplementary Figure 5 Impaired ratio compared based on whether groups receive chemotherapy **(A)** and radiotherapy **(B)** or not. We used Chi-squared test to compare between the groups and found no significant differences. Red, impaired group; gray, un-impaired group.
