# Supplementary material for: Simultaneous Damage of the Cingulate Cortex Zone II and Fronto-Striatal Circuit Causes Prolonged Selective Attentional Deficits
Source: Front Hum Neurosci. 2021 Dec 24;15:762578. doi: 10.3389/fnhum.2021.762578 (PMC8740164; doi:10.3389/fnhum.2021.762578)
Supplement: Supplementary file 1 [file Table_1.DOCX]

**Supplementary Table 1**. Details of attention functional score

| **Age** | **WHO grade** | **Tumor location** | **Impaired/un-impaired group** | | | **Letter cancellation test** | | | **Stroop test** | | |
| --- | --- | --- | --- | --- | --- | --- | --- | --- | --- | --- | --- |
|  |  |  | Pre op | 1 week | 3 months | Pre op | 1 week | 3 months | Pre op | 1 week | 3 months |
| 51 | 2 | frontal | 0 | 0 | 0 | 0 | 0 | 0 | 1 | 0 | 0 |
| 63 | 2 | frontal | 1 | 0 | 0 | 1 | 0 | 0 | 1 | 1 | 1 |
| 72 | 2 | frontal | 0 | 0 | 0 | 1 | 1 | 1 | 0 | 0 | 0 |
| 68 | 3 | frontal | 1 | 0 | 0 | 1 | 0 | 0 | 1 | 1 | 1 |
| 34 | 2 | frontal | 1 | 0 | 1 | 1 | 0 | 1 | 1 | 1 | 1 |
| 61 | 3 | frontal | 1 | 0 | 1 | 1 | 0 | 1 | 1 | 1 | 1 |
| 21 | 2 | parietal | 1 | 1 | 1 | 1 | 1 | 1 | 1 | 1 | 1 |
| 31 | 2 | frontal | 1 | 1 | 1 | 1 | 1 | 1 | 1 | 1 | 1 |
| 31 | 2 | frontal | 1 | 1 | 1 | 1 | 1 | 1 | 1 | 1 | 1 |
| 32 | 2 | temporal | 1 | 1 | 1 | 1 | 1 | 1 | 1 | 1 | 1 |
| 37 | 2 | frontal | 1 | 1 | 1 | 1 | 1 | 1 | 1 | 1 | 1 |
| 38 | 2 | frontal | 1 | 1 | 1 | 1 | 1 | 1 | 1 | 1 | 1 |
| 38 | 2 | frontal | 1 | 1^*^ | 1 | 1 | 1 | 1 | 1 | 1 | 1 |
| 41 | 2 | frontal | 1 | 1 | 1 | 1 | 1 | 1 | 1 | 1 | 1 |
| 41 | 2 | frontal | 1 | 1^*^ | 1 | 1 | 1 | 1 | 1 | 1 | 1 |
| 43 | 2 | frontal | 1 | 1 | 1 | 1 | 1 | 1 | 1 | 1 | 1 |
| 47 | 2 | frontal | 1 | 1 | 1 | 1 | 1 | 1 | 1 | 1 | 1 |
| 52 | 2 | temporal | 1 | 1 | 1 | 1 | 1 | 1 | 1 | 1 | 1 |
| 53 | 2 | temporal | 1 | 1 | 1 | 1 | 1 | 1 | 1 | 1 | 1 |
| 57 | 2 | frontal | 1 | 1^*^ | 1 | 1 | 1 | 1 | 1 | 1 | 1 |
| 59 | 2 | frontal | 1 | 1 | 1 | 1 | 1 | 1 | 1 | 1 | 1 |
| 61 | 2 | frontal | 1 | 1 | 1 | 1 | 1 | 1 | 1 | 1 | 1 |
| 65 | 2 | temporal | 1 | 1 | 1 | 1 | 1 | 1 | 1 | 1 | 1 |
| 71 | 2 | frontal | 1 | 1^*^ | 1 | 1 | 1 | 1 | 1 | 1 | 1 |
| 36 | 3 | temporal | 1 | 1 | 1 | 1 | 1 | 1 | 1 | 1 | 1 |
| 37 | 3 | frontal | 1 | 1 | 1 | 1 | 1 | 1 | 1 | 1 | 1 |
| 39 | 3 | frontal | 1 | 1 | 1 | 1 | 1 | 1 | 1 | 1 | 1 |
| 44 | 3 | frontal | 1 | 1 | 1 | 1 | 1 | 1 | 1 | 1 | 1 |
| 44 | 3 | frontal | 1 | 1 | 1 | 1 | 1 | 1 | 1 | 1 | 1 |
| 47 | 3 | frontal | 1 | 1 | 1 | 1 | 1 | 1 | 1 | 1 | 1 |
| 47 | 3 | parietal | 1 | 1 | 1 | 1 | 1 | 1 | 1 | 1 | 1 |
| 51 | 3 | parietal | 1 | 1 | 1 | 1 | 1 | 1 | 1 | 1 | 1 |
| 53 | 3 | frontal | 1 | 1 | 1 | 1 | 1 | 1 | 1 | 1 | 1 |
| 59 | 3 | frontal | 1 | 1 | 1 | 1 | 1 | 1 | 1 | 1 | 1 |
| 64 | 3 | frontal | 0 | 1^*^ | 1^*^ | 0 | 1 | 1 | 1 | 1 | 1 |
| 66 | 3 | frontal | 1 | 1 | 1 | 1 | 1 | 1 | 1 | 1 | 1 |

* Patients who are included in the un-impaired group in current study but are impaired when compared to age-matched controls. 0, impaired; 1, un-impaired.

**Supplementary Table 2**. Other neurological and cognitive function except for selective attention

| Factor | Number of patients (impaired/un-impaired) or Z-score | | |
| --- | --- | --- | --- |
|  | Non-resected | Resected | p-value |
| Movement | 1/25 | 2/8 | 0.12 |
| Sensation | 0/26 | 0/10 | NA |
| General cognitive function | -0.15 ± 1.20 | -0.86 ± 1.29 | 0.09 |
| Working memory | -0.15 ± 0.82 | -0.028 ± 1.17 | 0.71 |
| Verbal fluency | 0.15 ± 0.95 | -0.86 ± 1.29 | 0.090 |
| Visuospatial cognition | -0.048 ± 1.23 | -1.12 ± 1.82 | 0.077 |
| Emotion recognition | 0.34 ± 1.22 | -0.079 ± 1.16 | 0.40 |

We used Brunnstrom’s recovery test to assess movement，and categorized as impaired if the paresis was present. Sensation was assessed both superficial sensation and deep sensation. We used following assessments to assess each cognitive function; General cognitive function for Mini-mental state examination, working memory for 2-back test, verbal fluency for verbal fluency test of semantic and phonemic words, emotion recognition for expression recognition test for adult. Wilcoxon test or Chi-square test were used for statistical analyses. NA, not applicable.
