# Supplementary figures and images for: Simultaneous Damage of the Cingulate Cortex Zone II and Fronto-Striatal Circuit Causes Prolonged Selective Attentional Deficits
Source: Front Hum Neurosci. 2021 Dec 24;15:762578. doi: 10.3389/fnhum.2021.762578 (PMC8740164; doi:10.3389/fnhum.2021.762578)

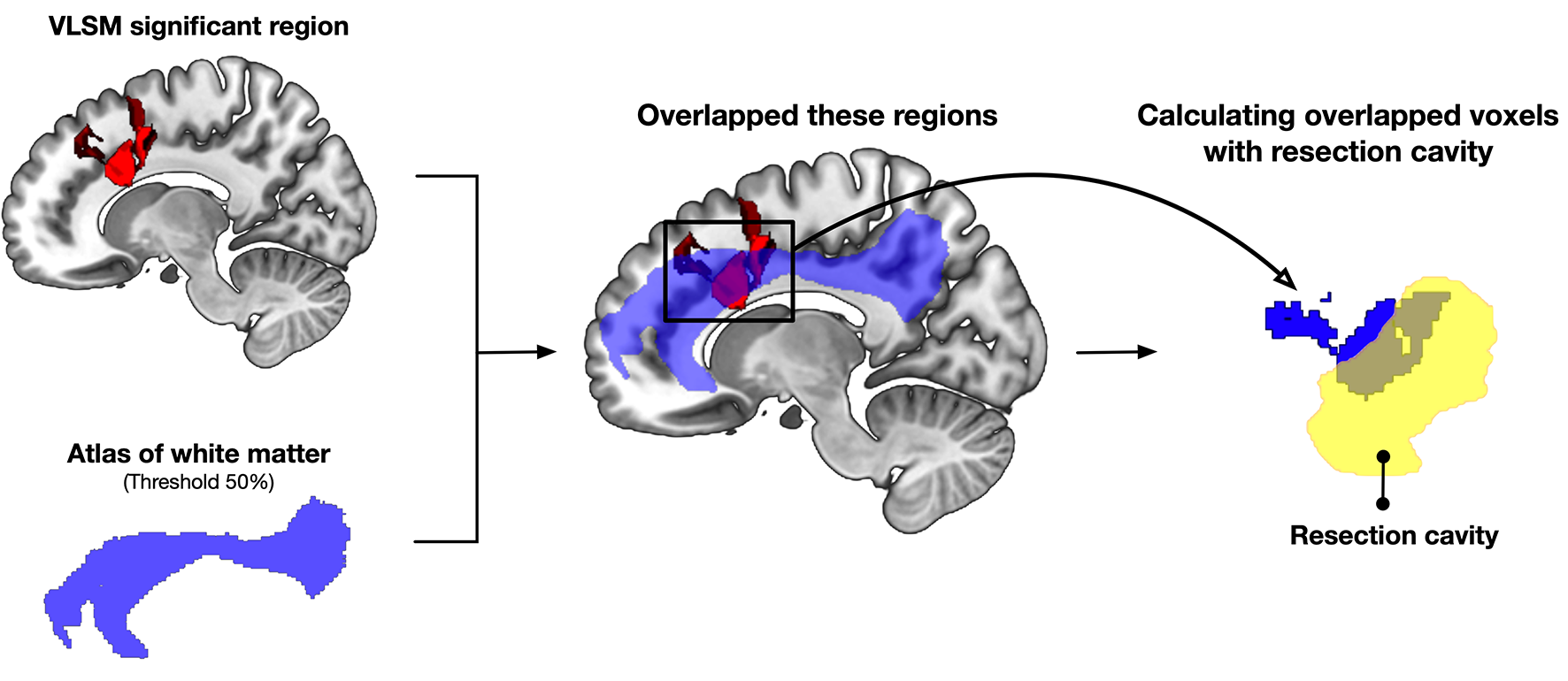

Supplement: Supplementary Figure 1 — A schema for calculating resected volume of white matter at the level of deep medial prefrontal cortex, namely voxel-based lesion-symptom mapping (VLSM) positive region. [file Image_1.TIFF]

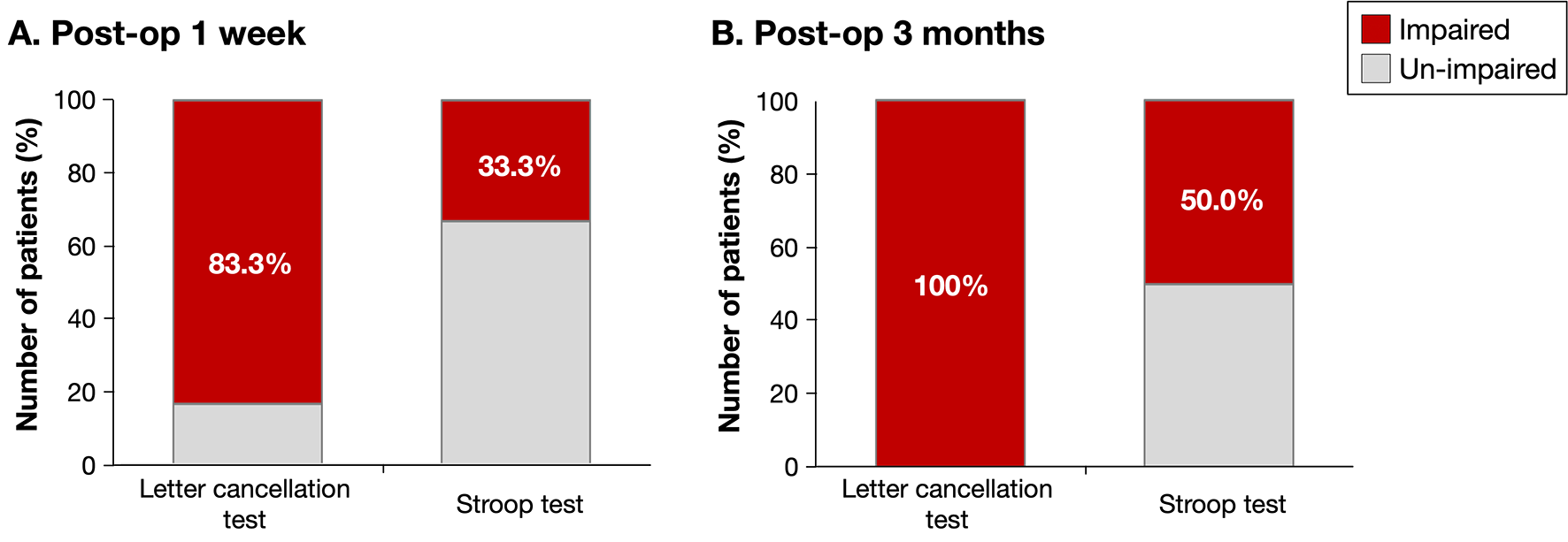

Supplement: Supplementary Figure 2 — Impaired ratio of selective attention in each test at post-op 1 week (A) and 3 months (B). Red, impaired (Z ≤ −1.65); Gray, un-impaired (Z > −1.65). [file Image_2.TIFF]

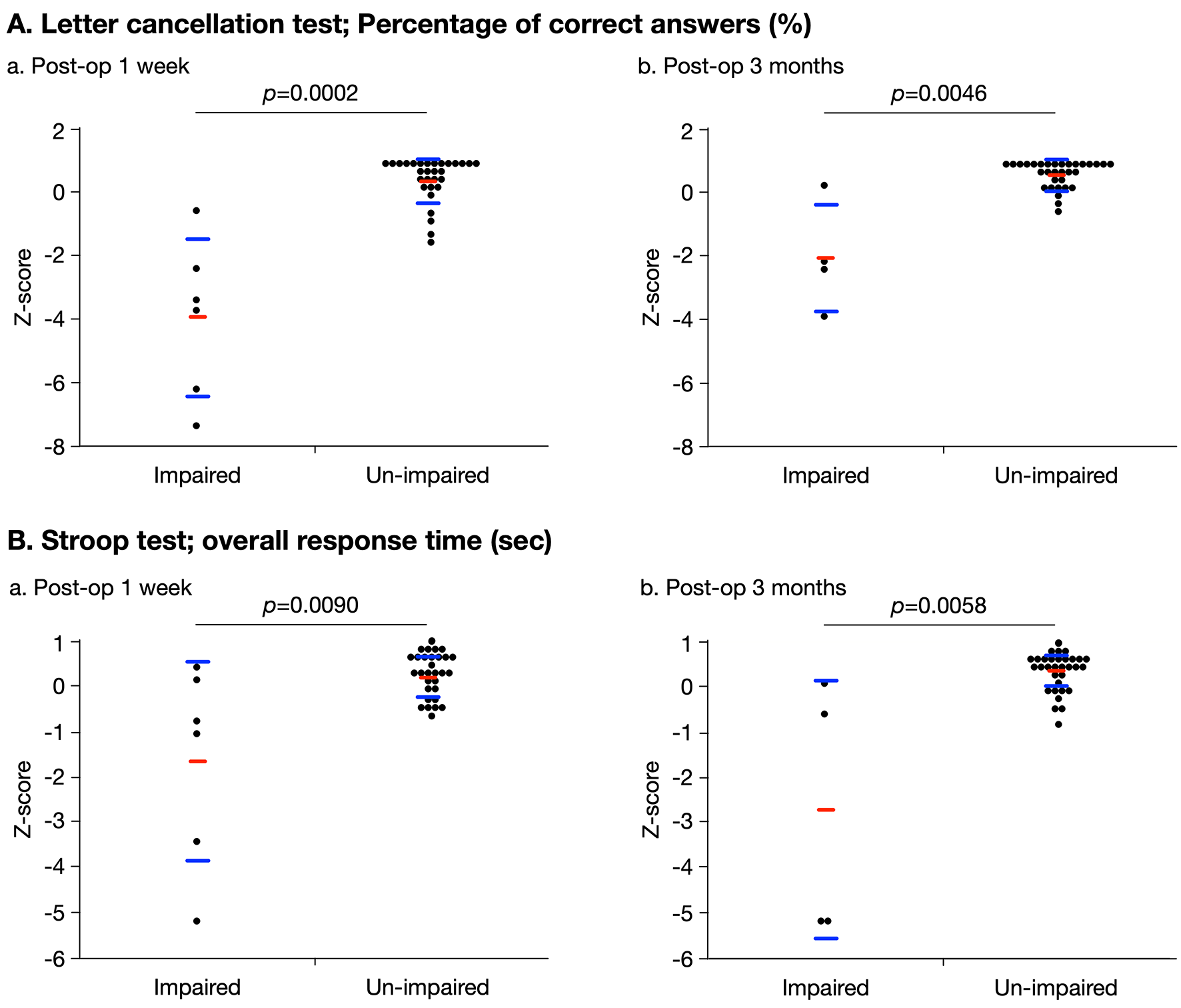

Supplement: Supplementary Figure 3 — Details of selective attention in each test at post-op 1 week (A) and 3 months (B). Wilcoxon test is used to compare the groups. Red line, mean value; Blue line, standard deviation. [file Image_3.TIFF]

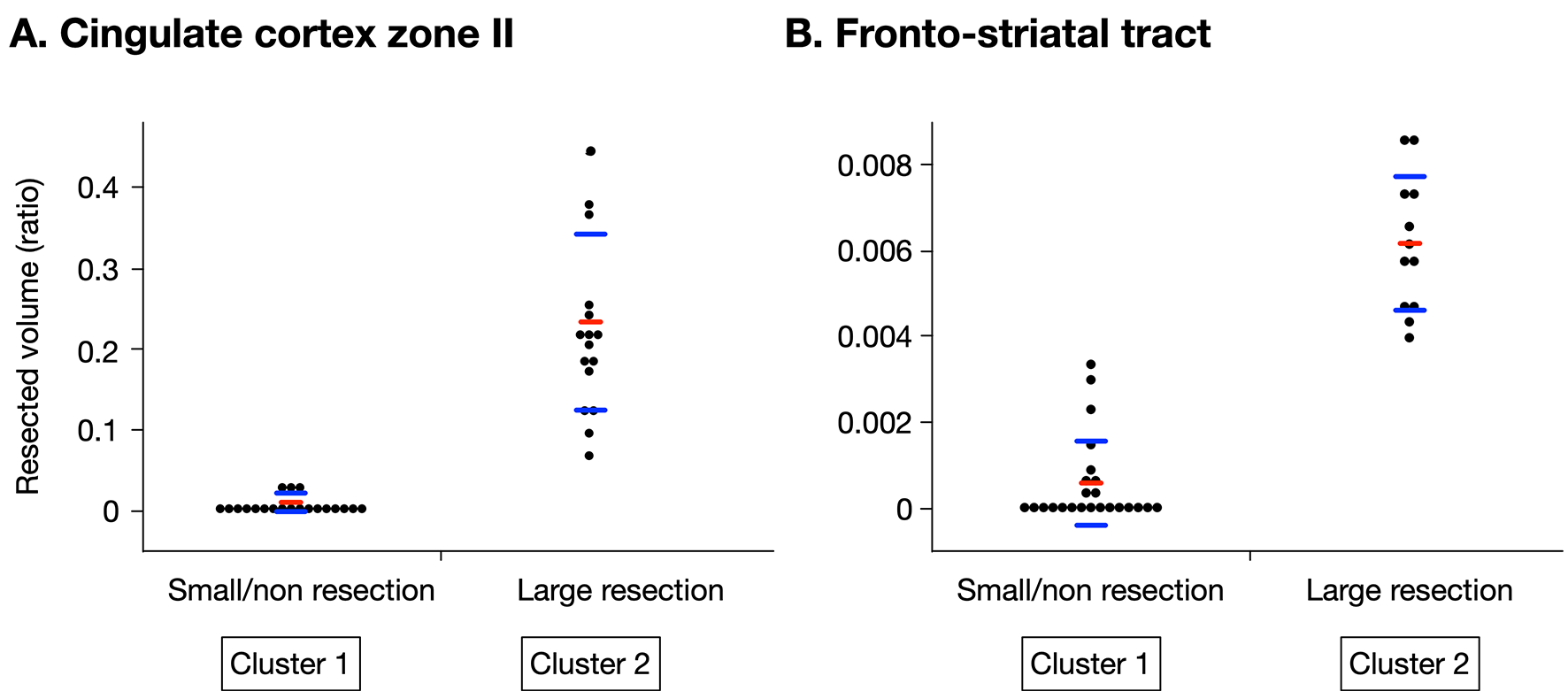

Supplement: Supplementary Figure 4 — Resected volume of the middle CC (A) and fronto-striatal tract (B) were divided into two groups using cluster analysis. We named cluster 1 as small/non-resection group, and cluster 2 as large resection group. Red, mean value; blue, standard deviation. [file Image_4.TIFF]

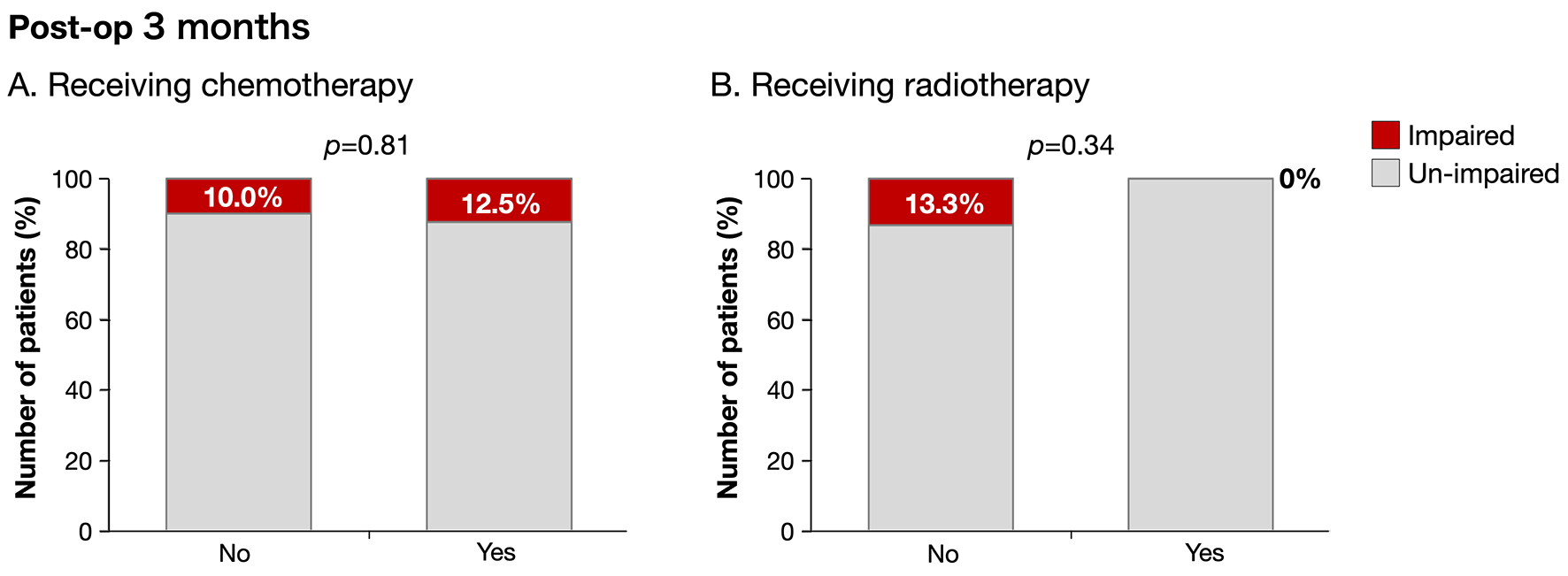

Supplement: Supplementary Figure 5 — Impaired ratio compared based on whether groups receive chemotherapy (A) and radiotherapy (B) or not. We used Chi-squared test to compare between the groups and found no significant differences. Red, impaired group; gray, un-impaired group. [file Image_5.TIFF]
